# Toward Dynamic Biomimetic Biomaterials: Temporal Coupling of Flavonoid-Induced Cellular Responses and 3D-Printed PLA Scaffold Evolution

**DOI:** 10.3390/biomimetics11090654

**Published:** 2026-09-11

**Authors:** Diana V. Portan, Panagiotis Zoumpoulakis, Vassilis Kostopoulos, Ioanna Pitterou, Konstantinos Tsiantas, Efstathios Michalopoulos, Leonard Azamfirei

**Affiliations:** 1 Laboratory of Biomechanics and Biomedical Engineering, Department of Mechanical Engineering and Aeronautics, University of Patras, 26504 Patras, Greece; portan@upatras.gr; 2 Faculty of Medicine, George Emil Palade University of Medicine, Pharmacy, Science and Technology of Targu Mures, 540142 Targu Mures, Romania; 3 Laboratory of Chemistry, Analysis & Design of Food Processes, Department of Food Science and Technology, University of West Attica, 12243 Egaleo, Greece; pzoump@uniwa.gr (P.Z.); ipitterou@uniwa.gr (I.P.); ktsiantas@uniwa.gr (K.T.); 4 Institute of Chemical Biology, National Hellenic Research Foundation, 48 Vas. Constantinou Ave., 11635 Athens, Greece; 5 Applied Mechanics Laboratory, Mechanical Engineering and Aeronautics Department, University of Patras, Rio Campus, 26500 Patras, Greece; kostopoulos@upatras.gr; 6 Hellenic Cord Blood Bank, Biomedical Research Foundation Academy of Athens, 4 Soranou Ephessiou Street, 11527 Athens, Greece; smichal@bioacademy.gr

**Keywords:** Wharton’s Jelly mesenchymal stem cells, naringin, hesperidin, polylactic acid, biomimetic scaffolds, bioactive compound delivery

## Abstract

**Background/Objectives**: Biomimetic biomaterials should be evaluated not only through their initial properties but also through their interaction with biological environments over time. This study investigated a human cell-based approach integrating flavonoid-induced cellular responses with the temporal evolution of 3D-printed polylactic acid (PLA) scaffolds under physiological-like conditions. **Methods**: Human Wharton’s Jelly mesenchymal stem cells (WJ-MSCs) were exposed to naringin and hesperidin (250 µg/mL) and evaluated after 3, 7, and 10 days using ALP, TP, OPN, and OC. In parallel, PLA scaffolds’ biodegradation was studied under static and dynamic conditions. **Results**: Naringin produced a more pronounced early/intermediate osteogenic-related response, whereas hesperidin showed comparatively more sustained ALP- and OPN-related activity at later stages. Dynamic scaffolds exhibited progressive temporal changes, with weight variations ranging from −0.384% to +0.127% at days 1, 3, 7, and 10, respectively, accompanied by progressive morphological modification. Initial AFM analysis showed an average RMS roughness of 22.1 nm. **Conclusions**: The distinct temporal profiles of the flavonoids, together with the evolving scaffold–medium interface, support a biomimetic strategy based on temporal coordination of biochemical cues. These findings provide a rationale for future experimentally validated sequential delivery systems designed to promote early osteogenic activation followed by sustained cellular and matrix-associated activity.

## 1. Introduction

The development of reliable human-relevant in vitro methodologies has become a major priority in pharmaceutical and biomedical research [1]. In line with the principles of the 3Rs (Replacement, Reduction and Refinement), increasing efforts are directed toward New Approach Methodologies (NAMs) capable of generating predictive and reproducible human-relevant data while reducing reliance on animal experimentation [2,3]. Primary human cell-based models are particularly valuable because they can respond to biochemical, physical, and material-derived cues within their surrounding microenvironment. However, the biological response of cells cannot be fully understood independently of the evolving environment in which they are cultured. Therefore, the development of human-relevant methodologies increasingly requires experimental approaches that consider cellular responses together with the physicochemical characteristics and temporal evolution of the surrounding microenvironment [4].

Among the bioactive compounds currently attracting considerable interest are naturally occurring flavonoids, which have been extensively investigated because of their antioxidant, anti-inflammatory, and tissue-regenerative properties [5,6]. Bioflavonoids constitute a major group of plant-derived polyphenols and are widely present in fruits and vegetables [7]. Citrus flavonoids naringin and hesperidin are particularly abundant and have been identified in citrus peels, pulp, juices, leaves, seeds, and flowers, with their concentration depending on plant variety, fruit maturation, and environmental conditions [8,9]. Their diverse biological activities have stimulated interest in pharmaceutical, nutraceutical, food, and other biomedical applications [10,11,12,13,14]. In particular, naringin and hesperidin have been associated with processes relevant to bone metabolism and tissue regeneration [15,16,17], making them suitable molecular stimuli for investigating time-dependent cellular responses in human primary cell-based models.

Nevertheless, despite the promising biological potential of flavonoids, their effects on human primary cells remain highly dependent on the experimental microenvironment and the temporal characteristics of exposure. Many studies focus on general characterization, isolated biological activities, or in vivo effects [5,6,7,8,9,10,11,17], whereas comparative investigations evaluating different bioactive molecules under identical human-cell-based conditions remain limited. Such comparative approaches are important for identifying compound-specific response patterns and establishing more reproducible NAM-based evaluation strategies. Moreover, when bioactive molecules are considered for incorporation into biomaterials, their biological effects should be interpreted together with the evolution of the material environment in which they are presented. This perspective shifts the evaluation of bioactive biomaterials from a static assessment of material properties toward a dynamic consideration of stimulus, cellular response, and material behavior.

Addressing this challenge, primary human cells isolated directly from donor tissues provide a more physiologically relevant model than conventional immortalized cell lines, responding to environmental stimuli such as substrate properties, bioactive compounds, and physical cues that contribute to the cellular microenvironment. This enables the assessment of cytocompatibility and bioactivity under human-relevant conditions that more closely reproduce key features of the cellular microenvironment [4]. As a result, primary cell-based in vitro models are increasingly recognized as an essential transitional step between laboratory research and clinical investigation, generating predictive human-relevant data while contributing to the reduction in animal experimentation [18,19,20]. The successful implementation of such human-relevant models also depends on biomaterial platforms capable of reproducing relevant cell–material interactions and, following appropriate validation, serving as carriers for localized bioactive compound delivery.

Regarding bioactive compound delivery, considerable efforts have been devoted to the development of advanced drug delivery systems capable of overcoming the intrinsic limitations of bioactive phytochemicals. Polymeric platforms, including hydrogels, nanofibers, nanoparticles and porous scaffolds, have been extensively investigated because they improve compound encapsulation, protect bioactive molecules from premature degradation, enhance bioavailability and provide controlled and localized release [21,22,23]. These approaches have been increasingly explored for the delivery of natural phytochemicals, including flavonoids, in regenerative medicine and implant-related applications, where controlled presentation of bioactive molecules within the local microenvironment is particularly relevant [24].

Recent advances in 3D printing, bioink development, and bioprinting technologies have further expanded biomaterial-based regenerative strategies by enabling the fabrication of constructs with controlled architectures designed to modulate cell–material interactions and reproduce selected features of the native tissue microenvironment. Digital light processing bioprinting approaches have demonstrated enhanced cellular proliferation within engineered hydrogel environments [25], while multifunctional printed scaffolds incorporating bioactive components and cells have shown promise for tissue reconstruction applications [26]. These developments highlight the importance of combining scaffold design based on biomimetic principles with reliable human-relevant evaluation methodologies to investigate cellular responses and scaffold behavior under physiologically relevant conditions.

Among the polymers available for additive manufacturing, polylactic acid (PLA) is one of the most extensively investigated because of its biocompatibility, biodegradability and ease of processing. Beyond their mechanical function, PLA scaffolds are increasingly being investigated as drug delivery platforms, where scaffold–medium interactions, fluid absorption, and the onset of polymer degradation are critical parameters governing drug-loading efficiency and release kinetics, while also influencing the evolving microenvironment surrounding the scaffold [27,28]. More recently, post-manufacture loading strategies have emerged as an attractive alternative to conventional pre-loading approaches for additively manufactured drug delivery systems. Instead of incorporating bioactive compounds into the polymer before filament extrusion or printing, therapeutic agents can be introduced after scaffold fabrication through solvent-assisted soaking or related surface loading techniques [29].

Although PLA-based systems and flavonoid-based therapeutic approaches have been extensively investigated independently, limited information is available regarding the relationship between compound-specific cellular responses and the simultaneous evolution of the polymeric scaffold microenvironment under physiologically relevant conditions. Understanding this dynamic interaction is an important step toward the rational design of future bioactive compound delivery systems based on biomimetic principles, particularly because both scaffold properties and cellular responses evolve over time.

Integrated in vitro approaches combining human cell responses with scaffold–medium interactions can provide a more comprehensive representation of the dynamic relationships established within a biomaterial-based microenvironment. Such approaches are particularly relevant to biomimetic research, where the objective is not only to evaluate the intrinsic biological activity of a compound or the structural properties of a scaffold, but also to understand how these components interact under conditions that reproduce selected features of the physiological environment. By integrating biological performance with material behavior, these experimental strategies can generate human-relevant datasets for the rational design and optimization of biomaterials intended for regenerative medicine and localized bioactive compound delivery, while supporting the development of advanced in vitro methodologies that may reduce reliance on animal models. This interdisciplinary perspective connects pharmaceutical sciences, biomaterial engineering, biomechanics, tissue engineering, and bioengineering.

The present study aimed to comparatively evaluate the biological responses of primary human Wharton’s Jelly mesenchymal stem cells (WJ-MSCs) to the citrus flavonoids naringin and hesperidin using a standardized human cell-based experimental approach. Specifically, the study investigated the time-dependent effects of the two flavonoids on cellular morphology, proliferation, alkaline phosphatase (ALP) activity, total protein (TP) content, ALP/TP ratio, and osteopontin (OPN) expression at defined incubation time points. In parallel, the study aimed to characterize the temporal evolution of 3D-printed PLA scaffolds under static and dynamic physiological-like immersion conditions by assessing weight variation and morphological changes. By examining these biological and material responses within complementary experimental components, the study sought to identify temporal patterns that may provide a basis for future biomimetic strategies integrating bioactive compounds with evolving scaffold microenvironments.

## 2. Materials and Methods

### 2.1. Preparation of Bioactive Compounds

Hesperidin (95%, catalog no. J62126) and naringin (≥95% by HPLC, catalog no. J63166), naturally occurring citrus flavanone glycosides of analytical purity, were purchased from Alfa Aesar (Ward Hill, MA, USA; now part of Thermo Fisher Scientific, Waltham, MA, USA).

Due to the limited aqueous solubility of naringin and hesperidin, stock solutions were prepared at a concentration of 50 mg/mL using dimethyl sulfoxide (DMSO) as an intermediate solvent. The compounds were initially dissolved in DMSO under gentle mixing until complete dissolution was achieved. For preparation of the treatment medium, 2.5 mL of the corresponding flavonoid stock solution was added to 497.5 mL of α-MEM supplemented with 10% fetal bovine serum (FBS), resulting in a final volume of 500 mL, a flavonoid concentration of 250 µg/mL, and a final DMSO concentration of 0.5% (*v*/*v*). The resulting solutions were sterilized by filtration through a 0.22 µm membrane under aseptic conditions prior to cell culture experiments.

A single concentration was selected to enable a direct comparison of the biological responses to the two flavonoids under identical experimental conditions. The selected concentration was chosen to be consistent with concentrations commonly used for bioactive supplementation in primary human mesenchymal stem cell cultures, where osteogenic supplements (e.g., L-ascorbic acid and other differentiation-inducing compounds) elicit measurable biological responses while maintaining cell viability.

### 2.2. Evaluation of Bioactive Compounds with Stem Cell Culture

WJ-MSCs were isolated from human umbilical cords (hUCs) obtained from the Hellenic Cord Blood Bank (HCBB), with cells derived from up to three donors whose cells were combined before the experimental procedures. Cell isolation was performed according to the protocol previously described by Chatzistamatiou et al. [30], in compliance with international ethical standards and local ethical regulations. Cells were maintained in α-MEM supplemented with 10% fetal bovine serum (FBS), 2 mM L-glutamine, 1% (*v*/*v*) amphotericin B, and 0.5% (*v*/*v*) gentamicin. Cells from the second and third passages were used for all experiments.

Cells were seeded in T25 flasks at a density of 4.0 × 10^4^ cells/cm^2^ and divided into three experimental groups: control, naringin-treated, and hesperidin-treated cultures. For each experimental condition and incubation time, three culture flasks were prepared from the same initial WJ-MSC culture and were therefore considered experimental replicates derived from a common cell population. Biomarker measurements were obtained from the corresponding cell lysates, with repeated assay measurements from the same lysate treated as technical replicates and averaged before statistical analysis.

Cell morphology and proliferation were monitored during the incubation period (3, 7, and 10 days) using a Leica DMI6000B inverted microscope (Leica Microsystems, Wetzlar, Germany) equipped with a 20× objective. Images were acquired and subsequently visualized and analyzed using ImageJ software v1.54.

Bioactive compound-treated cultures received naringin or hesperidin supplementation at a final concentration of 250 µg/mL. Cell responses were evaluated after 3, 7, and 10 days of incubation.

Alkaline phosphatase (ALP) activity was determined in cell lysates using an ALP enzyme activity assay kit (P0757L, BioLabs, Frankfurt am Main, Germany), according to the manufacturer’s instructions. Absorbance was measured at 405 nm. Total protein (TP) content was determined using the Pierce™ Coomassie (Bradford) Protein Assay Kit (Thermo Fisher Scientific, Waltham, MA, USA). Cell lysis for protein quantification was performed using 0.5% Triton X. Absorbance measurements were performed using an Infinite F200PRO UV/visible spectrometer (Tecan Austria GmbH, Grödig, Austria), with detection at 595 nm for total protein quantification. Osteocalcin (OC) and osteopontin (OPN) levels were quantified using human-specific ELISA kits (Wuxi Donglin Sci & Tech Development Co., Ltd., Wuxi, China) (DLDEVELOP, DLR-OC-Hu and DLR-OPN-Hu, respectively) according to the manufacturer’s instructions. For ELISA analysis, cells were detached by trypsinization and collected by centrifugation (10 min, 1700 rpm) in 15 mL tubes. Total protein (TP) content was determined from the same cell lysates used for the corresponding biomarker measurements and was used for normalization of ALP, OPN, and OC values, with the results expressed as ALP/TP, OPN/TP, and OC/TP, respectively. This normalization was used to account, at least partially, for differences in cellular biomass between samples and to reduce the potential influence of variations in cell number on biomarker measurements.

For ELISA measurements, three aliquots were collected from each experimental condition and analyzed in parallel. These measurements represent technical replicates obtained from the same cell culture condition. Data processing was performed using Origin software 2024 (Version 10.1), and results are presented as mean values with standard deviation (SD) and coefficient of variation (CV). As the present study was designed as an exploratory comparative investigation, biomarker data were evaluated descriptively using mean values, standard deviation, and coefficient of variation. No inferential statistical testing, effect-size estimation, or confidence-interval analysis was performed.

### 2.3. Fabrication and Observation of 3D-Printed Scaffolds

Three-dimensional cubic scaffolds (1 × 1 cm^3^) were printed with a 3DISON AEP desktop 3D printer (ROKIT, Seoul, Republic of Korea) using Fused Filament Fabrication (FFF). Natural-PLA (polylactic acid) with a diameter of 1.75 mm from Innofil3D (Emmen, The Netherlands) was used. Scaffolds were design as described in previous investigations of the authors, with square-shaped pores and a side length of 100 μm; the layers were perpendicular to each other (0°/90°), and their dimensions were 10 × 10 × 5 mm^3^. The scaffold porosity (39.16%) was calculated from the computer-aided design (CAD)/printing software (Ultimaker Cura software v4.4.11) based on the designed architecture and printing parameters, and based on our previous findings, their stiffness was around 177 MPa. The evolution of the mechanical behavior of PLA scaffolds after immersion under static and dynamic conditions has been investigated using dynamic mechanical analysis (DMA), demonstrating time-dependent changes in storage modulus during degradation [31].

The surface morphology and architecture of the 3D-printed PLA scaffolds were examined using a JSM-6610 scanning electron microscope (JEOL, Tokyo, Japan). Prior to imaging, the samples were gold-coated using a sputter coater (Bal-Tec SCD005, Balzers, Liechtenstein). SEM micrographs were acquired under high-vacuum conditions at an accelerating voltage of 2 kV and at different magnifications. Samples were analyzed at different magnifications. The surface roughness of the printed PLA material was additionally evaluated by atomic force microscopy (AFM) using a Nanoscope IIIa instrument (Veeco, CA, USA). Measurements were performed on multiple locations of non-immersed PLA samples to account for local surface variations, and the mean roughness value was calculated.

### 2.4. Experimental Setup for Scaffold Immersion and Weight Loss/Gain Evaluation

To evaluate the potential of the scaffolds to interact with bioactive compound-containing solutions, an investigation was carried out to assess scaffolds’ weight variation. The percentage of weight variation after immersion of the scaffolds in cell culture medium was calculated.

The immersion medium consisted of Alpha Minimum Essential Medium (α-MEM) supplemented with 10% fetal bovine serum (FBS), 50 µg/mL amphotericin B, 25 µg/mL gentamicin, and 50 µg/mL L-ascorbic acid. The immersion experiments were performed for 1, 3, 7 and 10 days under static and dynamic conditions, and the percentage of weight variation was calculated according to Equation (1):

(1) $$Weight\;change\;\%\;=\;\frac{Wf-Wi}{Wi}\;\times \;100$$

where Wi and Wf represent the initial and final scaffold weights, respectively.

The immersion experiment was designed to evaluate fluid uptake within the printed fibers at physiological body temperature (37 °C) under two parallel conditions: (a) a static condition, in which samples remained at the bottom of a glass beaker containing cell culture medium on a heating plate, and (b) a dynamic condition, in which samples were immersed under continuous stirring using a magnetic stirrer and heating plate at 500 rpm. The selected stirring speed was chosen to provide continuous fluid movement, medium renewal, and enhanced mass transport around the scaffold surface, thereby promoting increased interaction between the polymer structure and the surrounding fluid. The 500 rpm stirring condition was not intended to reproduce a specific physiological shear stress quantitatively, as the local shear stress generated by magnetic stirring depends on the vessel geometry, fluid properties, scaffold position, and resulting flow field. This dynamic condition was used as a simplified representation of aspects of the physiological fluid environment, where continuous fluid movement contributes to mass transport and exchange at the biomaterial–medium interface. Previous studies have demonstrated that capillary blood flow velocities vary considerably among vessels, with mean values ranging approximately from 0.2 to 2.7 mm/s, while also exhibiting temporal fluctuations. Therefore, the stirring condition was selected as an experimental approach to introduce continuous fluid movement and enhanced mass transport around the scaffold, allowing the investigation of scaffold–medium interactions under conditions more dynamic than static immersion, rather than as a quantitative simulation of physiological shear stress [32].

Three independent scaffold samples were analyzed for each experimental condition and immersion time point.

## 3. Results

### 3.1. Optical Microscope Observation

**Figure 1:**
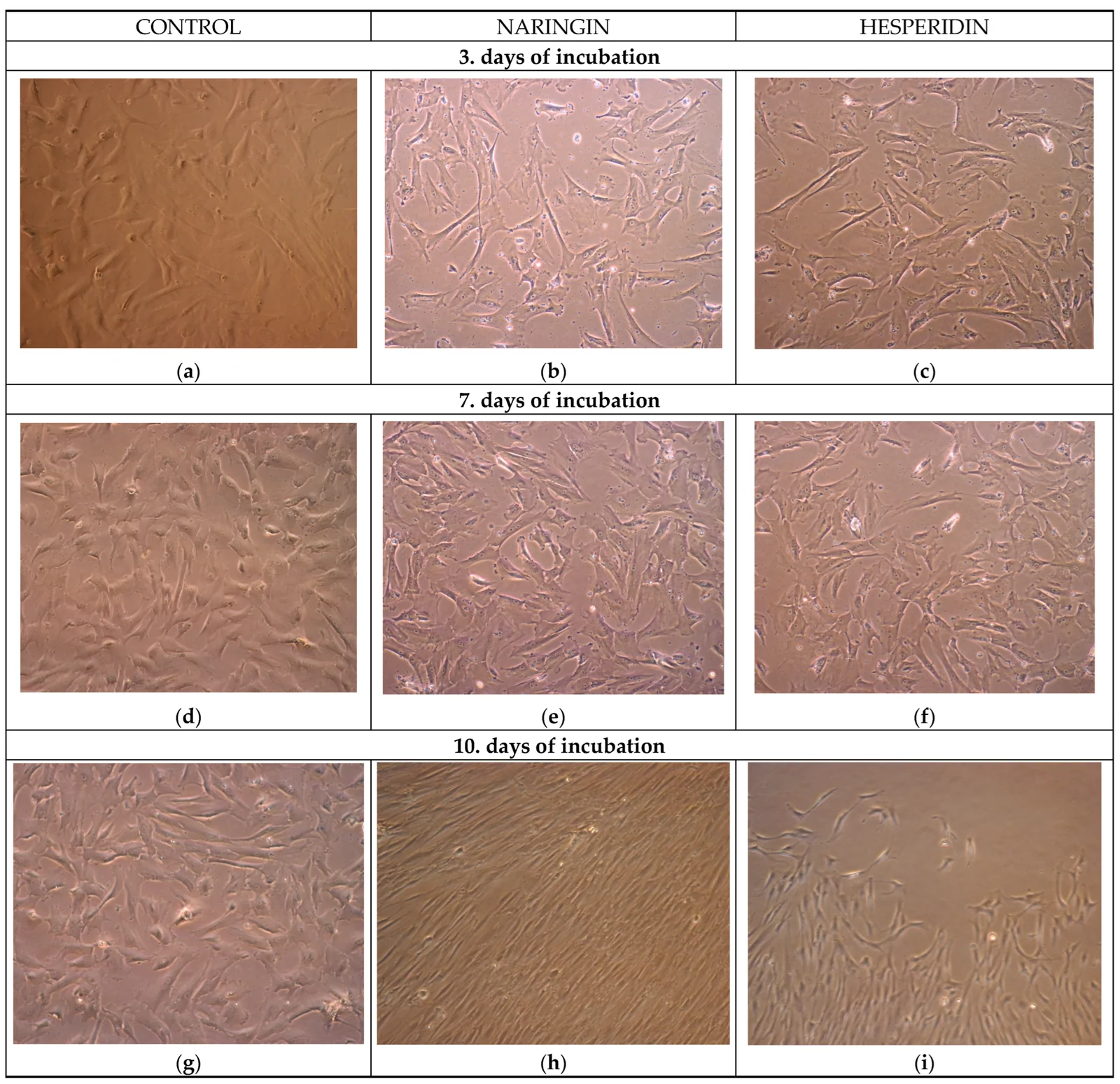
Optical microscopy images of the cells, magnification 20X: (**a**) column 1—control cultures after 3 days of incubation, (**d**) column 1—control cultures after 7 days of incubation, (**g**) column 1—control cultures after 10 days of incubation, (**b**) column 2—naringin-treated cultures, after 3 days of incubation, (**e**) column 2—naringin-treated cultures, after 7 days of incubation, (**h**) column 2—naringin-treated cultures, after 10 days of incubation (**c**) column 3—hesperidin-treated cultures after 3 days of incubation, (**f**) column 3—hesperidin-treated cultures after 7 days of incubation, (**i**) column 10—hesperidin-treated cultures after 10 days of incubation.

Microscopic observation of cell populations under defined culture conditions provides valuable information regarding cellular morphology, development, and changes in cell coverage over time. When combined with the analysis of specific biological markers, morphological observations can contribute to the interpretation of cellular responses to different microenvironmental stimuli. Figure 1 presents optical microscopy images of the cells at different incubation time points in the absence and presence of bioactive compounds. The left column (Figure 1a,d,g) shows stem cells maintained in standard culture medium after 3, 7, and 10 days of incubation.

As observed, the cells exhibited the typical morphology associated with this cell type [33]. Compared with untreated stem cells, cultures exposed to bioactive compounds (columns 2 and 3) displayed a slightly altered morphology. A more elongated, spindle-like morphology was observed in the cells, with a needle-like appearance particularly evident in Figure 1h. This morphological observation is further correlated with biomarker analysis in the Section 4.

In all experimental groups, progressive increases in cell coverage and culture confluence were observed from day 3 to day 10 of incubation. However, flasks with cells cultured in the presence of hesperidin exhibited areas of reduced cellular coverage still visible after 10 days of incubation compared with the control and naringin-treated groups. The analysis of the biomarkers elucidates this phenomenon.

### 3.2. WJ-MSCs and Osteogenic Biomarker Analysis

The levels of the osteogenic biomarkers alkaline phosphatase (ALP), total protein (TP), osteopontin (OPN), and osteocalcin (OC) were measured for all experimental groups after 3, 7, and 10 days of incubation. Representative values are presented in this section as both absolute values and, where applicable, values normalized to total protein (TP) for ALP, OPN, and OC. Normalization was performed to account for differences in cellular protein content among samples and to facilitate comparisons across treatment groups and incubation periods, considering that ALP is an early marker of osteogenic differentiation, whereas OPN and OC are associated with more advanced stages of osteogenesis.

#### 3.2.1. Alkaline Phosphatase and Total Protein

**Figure 2:**
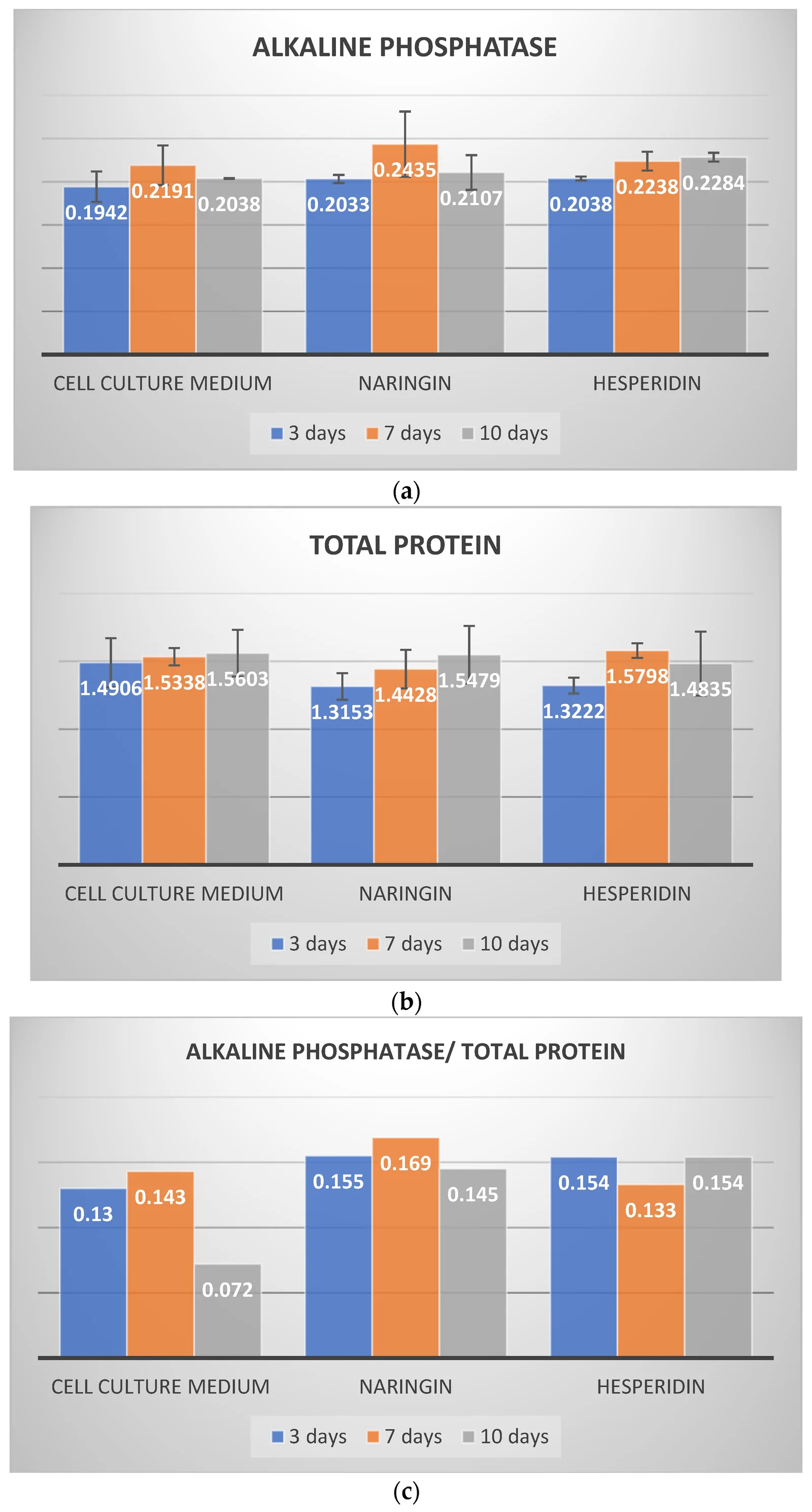
Biomarker levels in control, naringin-treated, and hesperidin-treated cell cultures: (**a**) alkaline phosphatase (ALP); (**b**) total protein (TP); and (**c**) alkaline phosphatase/total protein ratio (ALP/TP).

Alkaline phosphatase (ALP) activity (Figure 2a) and total protein (TP) content (Figure 2b) were evaluated after 3, 7, and 10 days of incubation, and ALP values normalized to TP content (ALP/TP ratio) were calculated (Figure 2c).

ALP activity exhibited a time-dependent variation among the experimental groups. In control cultures, ALP activity increased from day 3 to day 7, followed by a slight decrease on day 10. Naringin-treated cultures showed a similar pattern, with the highest ALP activity observed on day 7. In contrast, hesperidin-treated cultures demonstrated a progressive increase in ALP activity throughout the incubation period, reaching the highest value on day 10 among the evaluated time points.

Total protein content showed a gradual increase over time in control and naringin-treated cultures, whereas hesperidin-treated cultures exhibited an increase up to day 7 followed by a slight reduction on day 10.

Normalization of ALP activity to TP content revealed differences in the relative osteogenic response among the experimental groups. The ALP/TP ratio increased up to day 7 in naringin-treated cultures, followed by a decrease on day 10. Control cultures exhibited a reduction in the normalized ratio at the final time point, whereas hesperidin-treated cultures maintained relatively stable ALP/TP values throughout the incubation period.

#### 3.2.2. Osteopontin and Osteocalcin

**Figure 3:**
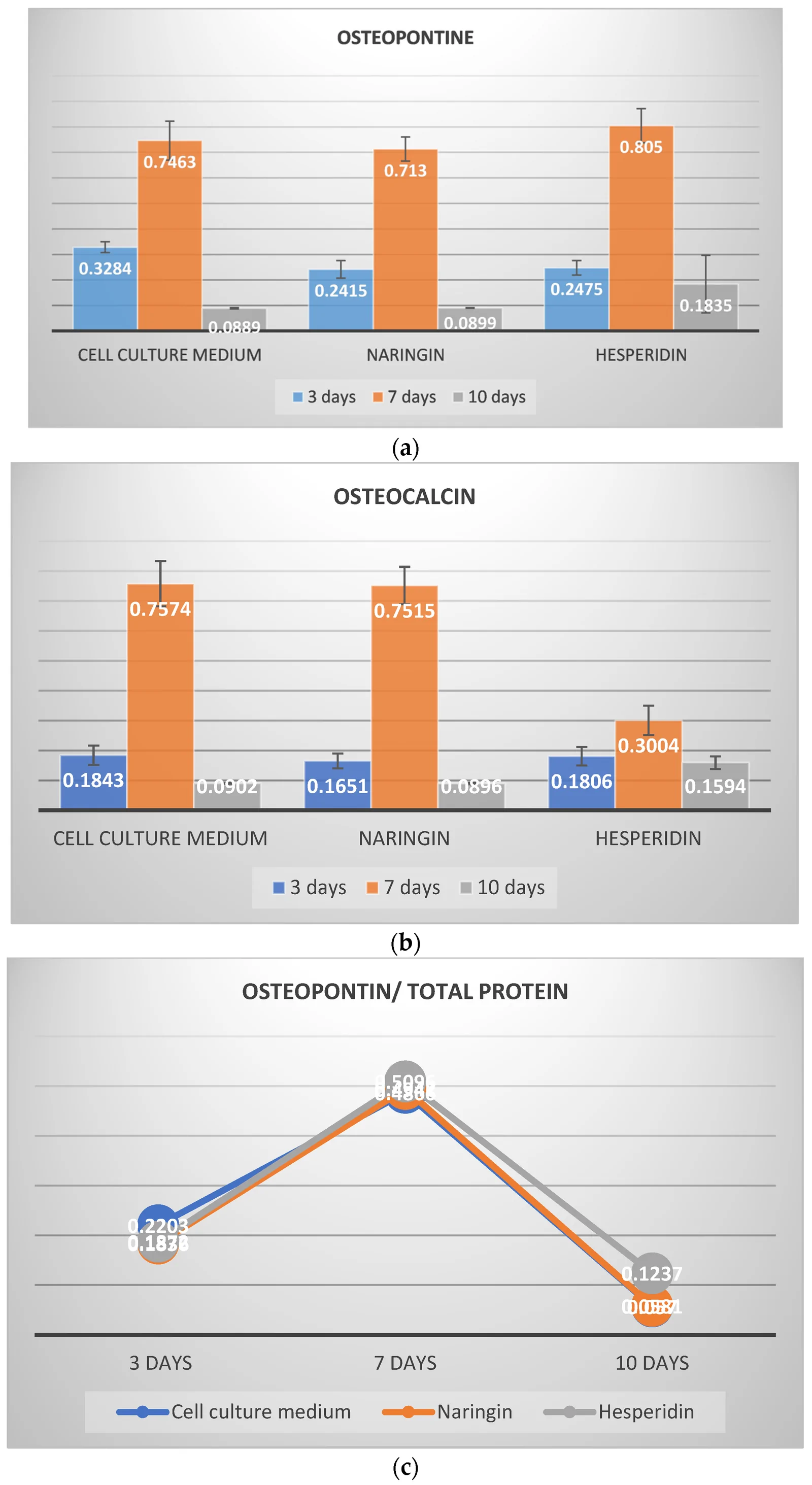

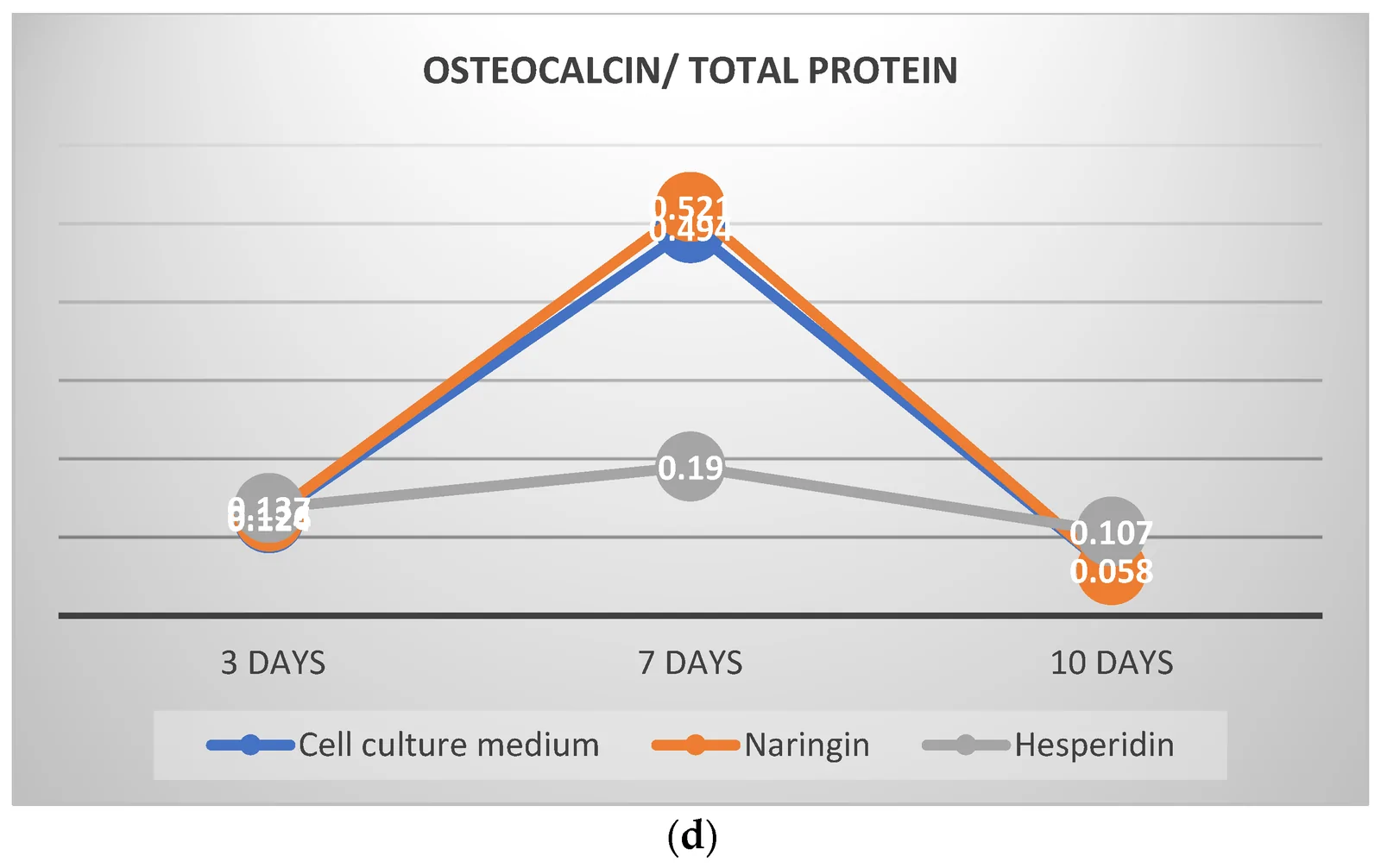
Biomarker levels in control, naringin-treated, and hesperidin-treated cell cultures: (**a**) osteopontin (OPN); (**b**) osteocalcin (OC); (**c**) osteopontin/total protein ratio (OPN/TP); and (**d**) osteocalcin/total protein ratio (OC/TP).

Osteopontin (OPN) and osteocalcin (OC) levels (Figure 3a,b) were evaluated after 3, 7, and 10 days of incubation, and the corresponding normalized values relative to total protein content (OPN/TP and OC/TP) were calculated (Figure 3c,d).

OPN levels showed a similar temporal profile among the experimental groups, with an increasing value from day 3 to day 7, followed by a pronounced decrease on day 10. The highest OPN values on day 7 were observed in hesperidin-treated cultures, while control and naringin-treated groups exhibited comparable responses. After normalization to TP, the OPN/TP ratio followed the same overall trend, with increased values on day 7 and decreased values on day 10, although hesperidin-treated cultures maintained higher normalized OPN levels at the final time point compared with the other groups.

OC levels also demonstrated a time-dependent variation, with increased values on day 7 followed by a reduction on day 10 in all groups. On day 7, control and naringin-treated cultures exhibited similar OC levels, whereas hesperidin-treated cultures showed lower values. The normalized OC/TP ratio reflected the same pattern, with a marked increase on day 7 in control and naringin-treated groups and a more moderate increase in the hesperidin-treated group. On day 10, hesperidin-treated cultures maintained higher OC/TP values compared with the other experimental groups.

### 3.3. Scaffold Immersion Results

#### 3.3.1. SEM Microscopy of Scaffolds

**Figure 4:**
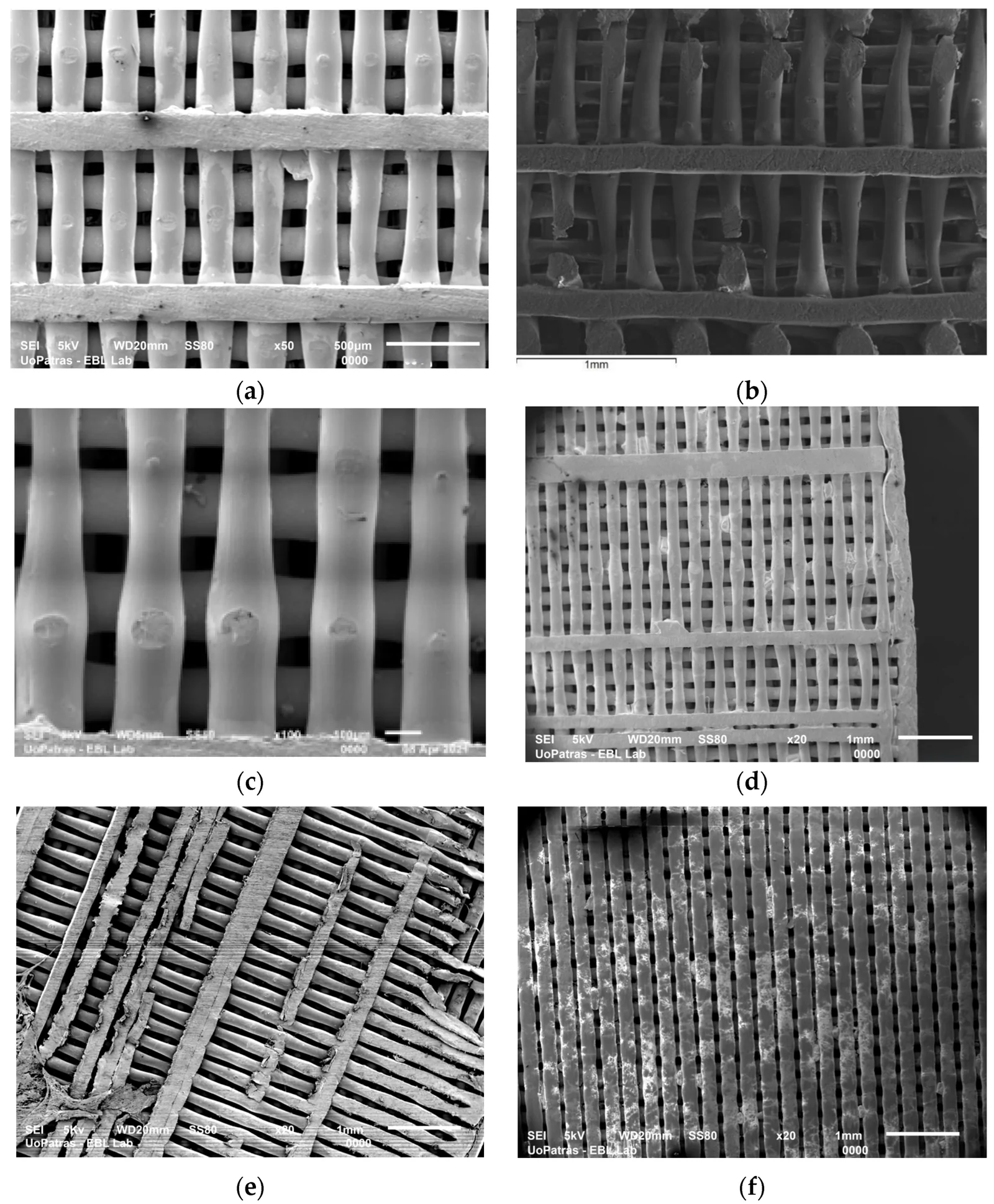
Representative scanning electron microscopy (SEM) images of PLA scaffolds: (**a**) control sample before immersion (day 0); (**b**) dynamic immersion after 1 day; (**c**) dynamic immersion after 3 days; (**d**) dynamic immersion after 7 days; (**e**) static immersion after 10 days; and (**f**) dynamic immersion after 10 days.

Selected SEM images of the scaffolds before and after immersion are presented in Figure 4. The control scaffold (Figure 4a) exhibited a highly organized architecture with a relatively smooth filament surface, while minor printing imperfections were also observed. Nevertheless, the overall structure appeared mechanically robust and well preserved.

During the immersion experiments, scaffolds maintained under static conditions did not exhibit pronounced morphological modifications during the initial stages of incubation. Although further analyses revealed early changes in scaffold weight, including an initial weight loss after the first day of immersion, these modifications were not clearly visible by SEM. This behavior is associated with localized surface phenomena, such as filament surface exfoliation, material delocalization, and the development of small cracks that were not sufficient to cause major structural disruption at the early stages. However, after 10 days of immersion, static samples displaying limited morphological alterations were observed (Figure 4e).

In contrast, scaffolds subjected to dynamic conditions exhibited more pronounced alterations, with visible filament breakage from the first day of immersion (Figure 4b), followed by regions of fiber thinning and surface deterioration consistent with increased material interaction under continuous agitation (Figure 4f).

The AFM measurements provide an additional characterization of the initial surface state of the printed PLA scaffolds, with an average RMS roughness of 22.1 nm and local values ranging from 9.455 to 34.988 nm. This spatial variation indicates that the printed surface is not topographically uniform at the nanoscale, which may contribute to local differences in cell–material interactions, including protein adsorption and subsequent cellular attachment. In the context of the present study, the AFM findings therefore complement the SEM and scaffold–medium interaction analyses by defining the initial surface characteristics of the biomaterial. Since AFM measurements were not performed longitudinally during immersion, the present data do not establish temporal changes in surface roughness; rather, they identify nanoscale surface heterogeneity as an inherent characteristic of the printed scaffold that may be relevant to the cellular microenvironment.

#### 3.3.2. Scaffold Weight Variation Measurements

The mean percentage of weight variation of the 3D-printed PLA scaffolds after immersion under static and dynamic conditions is presented in Table 1.

**Figure 5:**
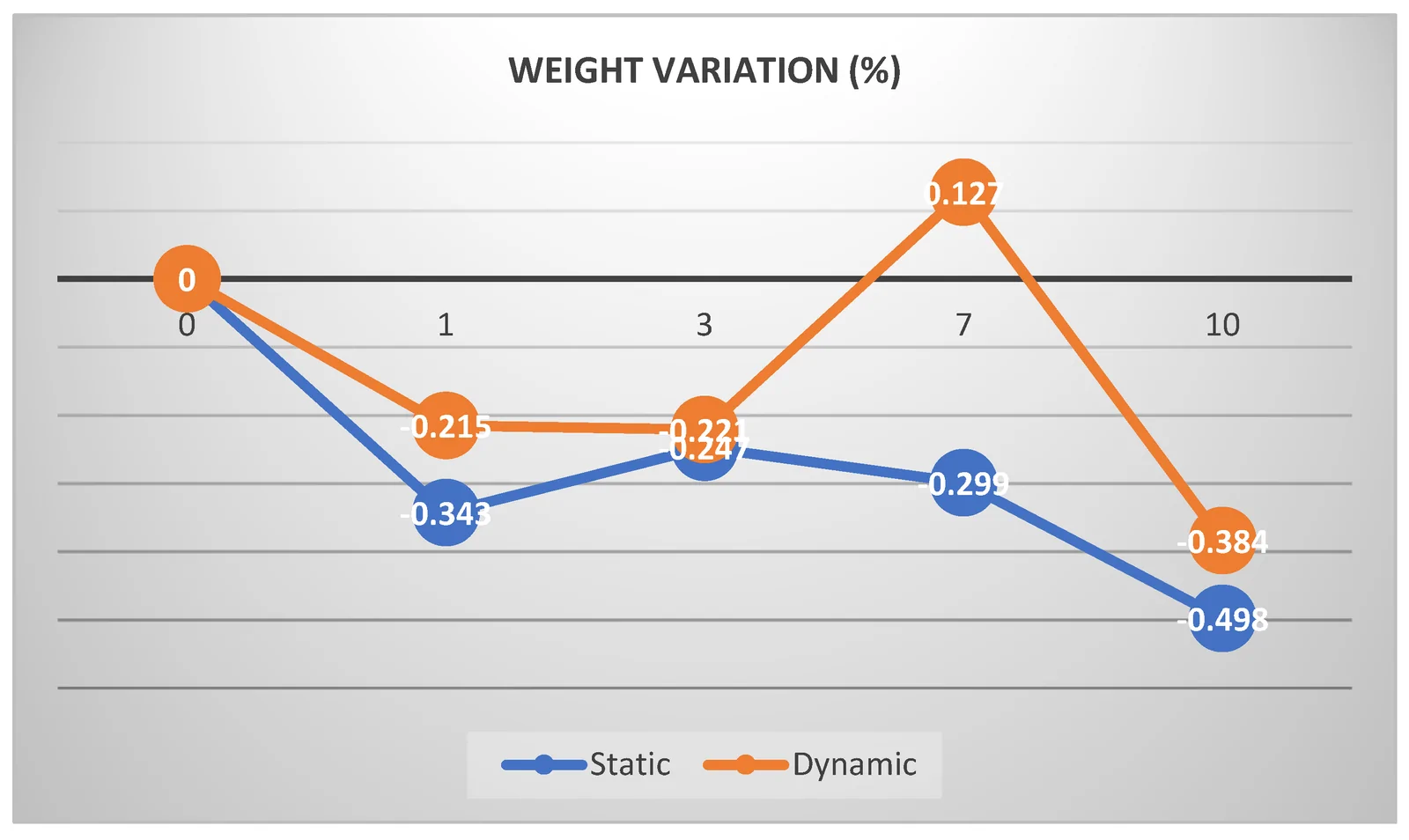
Weight variation of 3D-printed PLA scaffolds during immersion under static and dynamic (stirring) conditions.

Overall, both experimental conditions exhibited relatively small changes in scaffold weight throughout the immersion period (Figure 5).

Under static conditions, the scaffolds showed a slight weight decrease at all evaluated time points, with the highest weight reduction observed after 10 days of immersion. In the dynamic condition, a similar trend was observed during the first 3 days, with a slight decrease in weight, whereas a small increase in weight was recorded after 7 days of immersion. At 10 days, the dynamic condition also resulted in a weight reduction compared with the initial scaffold weight.

The percentage weight variation remained within a narrow range for both immersion conditions, with differences observed in the temporal pattern between static and dynamically immersed samples.

## 4. Discussion

### 4.1. Discussion on Cell Culture Response to Bioactive Compounds

The combined evaluation of multiple biomarkers is essential for understanding the complex biological processes occurring within a human-relevant in vitro microenvironment. Although the temporal evolution of individual biomarkers may not fully reflect the underlying cellular dynamics, the integrated analysis of complementary biomarkers provides a more comprehensive picture of the cellular responses induced by bioactive compounds and microenvironmental conditions. Furthermore, in primary cell cultures containing relatively small cell populations, even modest yet coordinated variations in multiple biomarkers may reflect biologically relevant responses that would be difficult to identify from a single parameter alone. From a biomimetic perspective, the combined assessment of cellular morphology, protein content, and osteogenic-related biomarkers provides a more comprehensive approach for evaluating how bioactive compounds modulate cell behavior within a controlled microenvironment.

Alkaline phosphatase is one of the key markers in the identification of pluripotent embryonic stem cells as well as related cells. Like other lineages and developmental-specific genes recognized as determinants of stem cells, the expression pattern of ALP during ontogenesis in some parts of tissues also corresponds with stem cell precursors and their niches. The key role of ALP is in the mineralization of hard tissue (it provides free phosphate for the creation of hydroxyapatite crystals and hydrolyzes pyrophosphate, an inhibitor of bone matrix formation) and in the metabolism of vitamin B6, and thus in the metabolism of neurotransmitter γ-aminobutyric acid (GABA). Therefore, the depletion or recessive mutation of ALP leads to defects in both the mineralization of hard tissue and the development of the nervous system [34]. However, this physiological interpretation represents the in vivo scenario, whereas in vitro cell culture systems require a more integrated analysis. Under controlled culture conditions, ALP variations may reflect changes in cellular activity, cellular development, and early differentiation processes occurring within the cell population. Therefore, when correlated with morphological observations and additional parameters such as total protein content, ALP provides valuable information regarding the overall health status, development, and functional response of the cultured cells.

As observed in the optical microscopy images (Figure 1), the control cultures exhibited progressive increases in cell coverage and confluence throughout the 10-day incubation period. This observation is expected, as in the absence of specific osteogenic differentiation factors, such as dexamethasone, primary stem cells generally maintain an active growth and maintenance phenotype under standard culture conditions. In contrast, as described in the Section 3, cultures exposed to naringin and hesperidin exhibited subtle morphological changes characterized by a more elongated and needle-like (Figure 1h) appearance compared with untreated cells. These morphological features are consistent with the typical characteristics reported for WJ-MSCs [33]; however, morphological observations alone were not considered sufficient to establish cellular differentiation and were therefore interpreted together with the biochemical biomarkers. Because the WJ-MSCs were cultured without conventional osteogenic induction supplements, the observed morphological changes were interpreted together with osteogenic-related biomarkers, such as ALP, OPN, and OC, to assess osteogenic-associated cellular activity rather than to demonstrate definitive osteogenic differentiation. Furthermore, Figure 1 shows that control and naringin-treated cultures exhibited progressive increases in cell coverage after 7 days of incubation, resulting in almost complete coverage of the flask surface by day 10. In contrast, hesperidin-treated cultures displayed lower cell coverage, with areas of uncovered surface still visible at the end of the incubation period. This observation may be related to the reported cytoprotective and antioxidant effects of hesperidin, which may influence cellular adaptation and functional maintenance under in vitro conditions [35]. Normalization of the biomarker values to total protein content partially accounts for differences in cellular biomass; however, because direct cell counting or an independent quantitative proliferation assay was not performed, a contribution of differences in cell number to the observed biomarker profiles cannot be completely excluded. In this context, the cellular microenvironment is influenced by available surface area, cell–cell interactions, substrate conditions, and the presence of bioactive compounds. These parameters collectively regulate cell behavior and may contribute to changes in proliferation and differentiation as culture progresses. Such interactions are relevant from a biomimetic perspective because they illustrate how cellular responses emerge from the dynamic relationship between cells, their surrounding microenvironment, and external biochemical cues.

### 4.2. Scaffold Weight Variation Interpretation

The second purpose of this study was to investigate the interplay between scaffold–medium interactions, fluid uptake, and early material modification processes in 3D-printed PLA scaffolds under physiological-like conditions. Particular attention was given to the temporal evolution of scaffold weight variation and the associated structural changes occurring during immersion. From a biomimetic perspective, the scaffold was considered not only as a structural material but as a dynamic interface interacting with its surrounding aqueous environment. The measured weight variation therefore reflects the combined effects of fluid penetration within the scaffold architecture and possible material loss associated with surface-related modifications and the release of soluble or loosely attached material fractions. Accordingly, the observed weight changes and SEM features were interpreted as evidence of time-dependent scaffold–medium interactions and material surface modification, rather than as direct quantitative measurements of PLA degradation. The extent of fluid uptake is influenced by the physicochemical properties of the thermoplastic material, its internal microstructure, and the characteristics of the surrounding medium. PLA degradation occurs primarily through hydrolysis of its ester bonds, while the degradation rate depends on environmental parameters such as temperature and pH, as well as on the intrinsic physicochemical properties of the polymer itself [36]. The experimental setup was designed to reproduce selected physiological-like conditions relevant to the potential implantation of PLA scaffolds. The immersion medium consisted of cell culture medium supplemented with serum and maintained at 37 °C, providing a controlled environment with a stable physiological pH suitable for evaluating scaffold–medium interactions. In addition, the dynamic immersion experiments introduced continuous fluid movement, enabling the assessment of scaffold behavior under enhanced mass transport conditions that reproduce certain aspects of the dynamic in vivo microenvironment compared with static incubation.

Upon immersion, the scaffolds are exposed to concurrent physicochemical processes, including fluid penetration into the porous architecture and polymer matrix, together with hydrolytic processes that progressively affect the material structure. These phenomena occur simultaneously and continuously influence one another, with their relative contribution varying over time. From a biomimetic perspective, this dynamic material evolution is relevant because the local microenvironment surrounding a scaffold is continuously modified by fluid transport, surface interactions, and changes in material properties. Fluid uptake temporarily contributes to increased scaffold weight, whereas hydrolytic processes and localized material loss gradually alter the structural integrity of the polymer. Understanding the balance between these competing processes may be relevant to the development of implantable drug delivery platforms, as changes at the scaffold–medium interface could potentially influence the loading, retention, and release of bioactive compounds while also affecting the stability and biological functionality of the printed structure.

In this study, in addition to SEM observations, AFM analysis provided further information regarding the surface characteristics of the printed PLA fibers. The variability observed among different AFM measurement locations reflects the heterogeneous nanoscale surface characteristics generated during the fused-filament fabrication process. From a biomimetic perspective, such surface heterogeneity is relevant because nanoscale topographical features can influence the interaction of the scaffold with its surrounding biological environment. These surface irregularities may contribute to increased interaction between the PLA fibers and the surrounding medium by providing additional nanoscale features for fluid contact and molecular adsorption. Consequently, the surface characteristics of the printed scaffold represent an important component of the scaffold–environment interface and may influence subsequent fluid penetration and bioactive compound interactions. The weight variation profile (Figure 5) indicates that both static and dynamic immersion conditions produced only minor changes in scaffold mass during the first 24 h. At this early stage, both groups exhibited a slight net weight loss, suggesting that fluid uptake and early material loss-related processes occurred simultaneously. Because the measured weight represents the balance between these competing phenomena, the gravimetric results do not allow the individual contribution of fluid uptake and material loss to be quantified. Nevertheless, the smaller weight reduction observed under dynamic conditions indicates that enhanced scaffold–medium interactions partially compensated for the initial mass loss. Continuous agitation promotes medium renewal around the scaffold surface and enhances mass transport, thereby facilitating fluid penetration into the porous architecture and increasing contact between the polymer structure and the surrounding medium. These dynamic transport conditions provide a more biomimetic representation of fluid movement than static immersion and may influence the evolution of the scaffold–medium interface during the early stages of exposure.

The cell culture medium used in the immersion experiments contains water, inorganic salts, and biological macromolecules, with fetal bovine serum (FBS) representing an important source of proteins. Serum albumin is the predominant protein component of FBS, accounting for approximately 60–67% of total serum proteins [37]. Upon immersion, the PLA scaffold is therefore exposed to a complex biological-like medium in which multiple interactions may occur simultaneously, including adsorption of proteins onto the polymer surface and penetration of aqueous components into the scaffold architecture. From a biomimetic perspective, the presence of serum proteins provides an additional level of complexity compared with purely aqueous immersion systems, allowing the scaffold–medium interface to be evaluated under conditions that more closely reflect the molecular environment encountered by biomaterials in biological fluids.

Protein adsorption onto biomaterial surfaces is governed by physicochemical interactions involving changes in interfacial free energy, which can be described through thermodynamic relationships such as Gibbs free energy:

*G* = *H* − *TS* (2)

*G* = *U* + *PV* − *TS* (3)

where U represents the internal energy of the system, P the pressure, V the volume, T the temperature, S the entropy, and H the enthalpy. Although the present study focuses primarily on fluid penetration within the scaffold fibers and porous structure, this phenomenon is closely associated with scaffold–medium interactions and is relevant for the future development of biomimetic drug delivery systems. Protein adsorption is therefore also considered, as the formation of a protein layer at the polymer surface may influence subsequent interactions between the PLA scaffold and the aqueous environment. Under dynamic conditions, the continuous movement of the immersion medium generated by magnetic stirring enhances convective transport and increases the contact between serum proteins and the scaffold surface. These interactions contribute to the formation of a dynamic scaffold–medium interface, an important consideration when designing biomaterials intended to reproduce selected features of the biological microenvironment.

The surface characteristics of PLA are strongly influenced by its relatively low wettability, although various surface and bulk modification strategies have been developed to increase hydrophilicity and improve biological interactions [38]. Nevertheless, the relatively hydrophobic nature of PLA does not prevent protein adsorption, as interactions between proteins and polymer surfaces are governed by multiple factors, including surface chemistry, wettability, protein conformation, and interfacial free energy. Hydrophobic interactions may promote the adsorption of proteins by favoring interactions between non-polar regions of proteins and the polymer surface, accompanied by the displacement of water molecules from the interface [39]. Therefore, unmodified PLA fibers provide a suitable surface for protein adsorption, while their intrinsic wettability characteristics may limit direct aqueous interaction at the polymer surface. From a biomimetic perspective, these surface properties are important because the physicochemical characteristics of the scaffold determine the nature of the initial interface established with the surrounding biological-like medium. However, in porous 3D-printed scaffolds, fluid penetration is also strongly influenced by the internal architecture, pore geometry, and interconnectivity of the structure, which are critical parameters for reproducing relevant aspects of the three-dimensional biological microenvironment and for future drug-loading and release applications.

The pressure conditions within the immersion system are influenced by the experimental configuration and the relative position of the scaffold within the surrounding medium. In the static setup, the scaffolds remained at the bottom of the container, whereas under dynamic conditions the continuous fluid movement altered their position and exposure to the surrounding medium. However, considering the relatively small differences in hydrostatic pressure expected within the experimental volume, pressure variations are unlikely to represent a major factor affecting the observed scaffold weight changes or surface interactions. The volume of immersion medium was kept constant in both experimental conditions (500 mL), and the temperature was controlled at 37 °C. These controlled parameters helped maintain a consistent experimental microenvironment, allowing the effects of fluid movement and scaffold–medium interactions to be evaluated more clearly.

The main difference between static and dynamic conditions arises from fluid movement and associated mass transport phenomena. Continuous agitation enhances medium circulation around the scaffold, increasing the exchange of molecules at the polymer–medium interface and promoting greater interaction between serum proteins and the PLA surface. Therefore, dynamic conditions favor a more active scaffold–medium interaction environment compared with static immersion. From a biomimetic perspective, fluid movement and mass transport represent important components of the dynamic microenvironment surrounding implanted biomaterials, and their inclusion provides a more physiologically relevant context for evaluating scaffold evolution and material–medium interactions.

The temporal evolution of scaffold weight variation indicates that scaffold–medium interactions developed progressively during immersion, with both fluid uptake and material loss contributing to the measured response. During the first 24 h, both static and dynamic conditions exhibited a slight reduction in scaffold mass, suggesting that early surface-related material loss occurred simultaneously with the initial penetration of aqueous components into the scaffold structure. These findings illustrate the dynamic nature of the scaffold–medium interface, in which fluid transport and early material modification occur concurrently. Such temporal changes are relevant to biomimetic scaffold design because the physicochemical properties of the material are not static after implantation but may progressively evolve in response to the surrounding biological environment.

During the following immersion period, the two experimental conditions exhibited different temporal profiles. Under dynamic conditions, the transition from initial weight loss to a slight weight increase on day 7 indicates a greater contribution of medium-derived components retained within the scaffold architecture, including possible accumulation of proteins or other dissolved molecules from the culture medium. Continuous agitation enhances mass transport and increases contact between the polymer surface and the surrounding medium, facilitating the exchange of molecules between the scaffold and its environment. Under static conditions, the scaffold maintained a continuous slight decrease in weight throughout the immersion period, suggesting that material loss-related processes gradually dominated over potential fluid uptake. The absence of fluid renewal and reduced convective transport may limit the accumulation of medium components within the scaffold structure, while prolonged exposure to the aqueous environment promotes gradual physicochemical changes of the PLA surface. The SEM observations after 10 days support the occurrence of structural modifications, including surface deterioration and filament damage. From a biomimetic perspective, these differences between static and dynamic conditions demonstrate how fluid transport can influence the evolving scaffold microenvironment and consequently the interaction between the material and its surrounding medium.

In both experimental conditions, the porous architecture of the 3D-printed scaffold remains a critical factor governing scaffold–medium interactions. Structural modifications, including localized surface erosion and filament damage, may alter the available surface area and accessibility of the internal pores, thereby influencing subsequent interactions with the surrounding medium. These observations emphasize the importance of considering scaffold architecture as a dynamic component of the biomimetic microenvironment, where changes in pore accessibility and surface characteristics may progressively modify fluid transport, molecular interactions, and the potential availability of bioactive compounds.

It should be noted that the present study evaluated scaffold behavior through morphological observations and gravimetric analysis. While the observed weight changes and SEM features indicate dynamic interactions between the PLA scaffold and the surrounding medium, they do not directly quantify polymer degradation. Further investigations involving chemical and molecular characterization techniques, such as FTIR, DSC, gel permeation chromatography, or degradation product analysis, would provide additional information regarding the mechanisms of PLA degradation during immersion. Such analyses would also help clarify how the evolving physicochemical properties of the scaffold contribute to the dynamic biomaterial–environment interface, an important consideration for the development of biomimetic scaffolds intended for regenerative medicine and localized bioactive compound delivery. Furthermore, wettability, protein adsorption, and bioactive compound availability were not directly measured in the present study; therefore, their potential contribution to the observed scaffold–medium interactions remains speculative and should be investigated in future studies.

### 4.3. Implications of Scaffold–Medium Interactions for Sequential Bioactive Compound Delivery and Cellular Modulation

The biological results obtained for naringin and hesperidin suggest that a sequential delivery strategy could be particularly relevant for biomimetic osteogenic applications. In the present study, naringin exposure resulted in a more pronounced early increase in osteogenic-related activity, as reflected by the ALP and ALP/TP profiles, together with increased OPN expression at intermediate incubation times. These findings indicate that naringin exerts a stronger influence during the early stages of cellular activation and matrix-associated development. Conversely, hesperidin exhibited a more prolonged response, maintaining higher ALP values at later incubation stages and presenting the highest OPN/TP values during the final time point. This temporal difference suggests that hesperidin may be associated with a more sustained extracellular matrix-related response during the later stages of incubation. From a biomimetic perspective, these distinct temporal responses highlight the potential value of designing biomaterial systems that do not provide a constant biochemical stimulus, but instead reproduce selected aspects of the sequential and evolving biochemical cues encountered during tissue development and regeneration.

To further integrate the material and biological findings, the temporal evolution of the dynamically immersed PLA scaffolds was considered alongside the time-dependent changes observed in the cellular biomarkers. This comparison does not imply a direct causal relationship between scaffold modification and cellular response; rather, it provides a temporal framework for identifying potential windows in which changes at the scaffold–medium interface coincide with distinct stages of cellular activity. As summarized in Table 2, the dynamic scaffold response evolves from early surface and filament modifications, through a stage characterized by increased medium interaction and transient mass retention, to more pronounced structural deterioration at day 10. These material events occur within the same temporal framework as the distinct biomarker trajectories induced by naringin and hesperidin, revealing a potentially relevant relationship between the evolving biomaterial microenvironment and the timing of cellular responses.

Taken together, the temporal relationship between scaffold evolution and cellular responses suggests a potential biomimetic programming strategy in which early scaffold–medium interactions could be associated with the availability of naringin during an early/intermediate phase of osteogenic activation, followed by prolonged hesperidin availability as the scaffold–medium interface evolves and cellular responses become more sustained. Such temporal organization could potentially reproduce sequential biochemical cues associated with cellular activation and subsequent matrix-associated activity. Importantly, this interpretation represents a design hypothesis derived from the temporal concordance of the present findings and does not demonstrate sequential compound release, which requires direct loading and release experiments.

The temporal modifications observed in the PLA scaffolds during immersion provide important considerations for their potential application as bioactive compound delivery platforms. The weight variation profile and SEM observations indicate that scaffold–medium interactions begin immediately after immersion and progressively modify the polymer surface and internal architecture. Changes consistent with fluid uptake or retention were observed between days 1 and 3 of immersion, mainly under static conditions, although gravimetric analysis cannot distinguish fluid uptake from concurrent material loss. From a biomimetic perspective, these findings indicate that the scaffold does not represent a static carrier, but a dynamic microenvironment whose physicochemical properties evolve over time in response to fluid exposure and scaffold–medium interactions. Such temporal evolution may be exploited in future designs to modulate the availability of bioactive compounds according to the changing requirements of the cellular microenvironment.

The progressive evolution of the PLA scaffold provides a conceptual basis for considering how scaffold architecture and scaffold–medium interactions could be exploited to modulate the temporal availability of bioactive compounds. The observed transition from early filament modification to increasing fluid interaction and transient medium retention, followed by more pronounced structural deterioration at day 10, suggests that the scaffold microenvironment may undergo distinct temporal states that could be incorporated into the design of future delivery systems. Rather than assuming that scaffold modification directly determines compound release, these observations support the development of spatially and temporally programmed architectures in which the localization, association, or loading of individual compounds could be deliberately controlled. Within such a framework, naringin could be positioned to provide an early/intermediate osteogenic stimulus, whereas hesperidin could be designed for prolonged availability during later stages of cellular response. This strategy would shift the design concept from simultaneous compound delivery toward temporal coordination between scaffold evolution and cellular requirements. Nevertheless, the present study provides only the biological and material rationale for such a strategy; the actual release behavior, compound-specific retention, and temporal sequence would require direct experimental investigation using loaded scaffolds and quantitative release studies.

In addition to their role as carriers, evolving scaffold properties directly influence the cellular microenvironment experienced by adherent cells. Changes in surface morphology, wettability, protein adsorption, and local biochemical composition can modify cell attachment, proliferation, and differentiation pathways by altering the interaction between the cellular membrane, extracellular matrix components, and the underlying biomaterial. Therefore, scaffold evolution and bioactive compound release should be considered as interconnected processes, in which material properties and biological signaling dynamically influence one another. From a biomimetic perspective, this reciprocal relationship is particularly relevant because native tissues provide cells with continuously changing biochemical, physicochemical, and mechanical cues rather than a static environment. Accordingly, the integration of temporal scaffold evolution with controlled bioactive signaling may provide a more physiologically relevant strategy for designing regenerative biomaterials.

## 5. Conclusions

This study applied a complementary human-relevant NAM-based approach to evaluate the biological effects of bioactive flavonoids and to investigate the interaction behavior of 3D-printed PLA scaffolds under physiological-like immersion conditions. By integrating primary human cell responses with the characterization of scaffold–medium interactions, the study provides a biomimetic framework in which biological activity and material evolution can be considered as interconnected components of the experimental microenvironment. This approach supports the development of more predictive in vitro strategies for investigating bioactive compounds and biomaterial systems intended for regenerative medicine and localized delivery applications.

The use of primary human Wharton’s Jelly mesenchymal stem cells provided a human-relevant in vitro model to investigate compound-specific cellular responses to naringin and hesperidin without relying on animal models. The biological evaluation revealed distinct temporal effects of the two flavonoids on WJ-MSC behavior. Naringin showed a more pronounced early osteogenic-related response, characterized by increased ALP activity, the highest ALP/TP ratio on day 7, and enhanced osteopontin expression at intermediate culture stages. In contrast, hesperidin promoted a more sustained cellular response, maintaining the highest ALP value on day 10 and exhibiting higher OPN and OPN/TP values during the later stages of incubation. These differences indicate that complementary biomarkers are required to characterize the complex, time-dependent cellular responses induced by bioactive compounds. They also highlight the importance of reproducing relevant biochemical and temporal cues when developing biomimetic in vitro models for regenerative medicine.

The evaluation of 3D-printed PLA scaffolds demonstrated that the printed structures undergo continuous scaffold–medium interactions under physiological-like conditions. Both static and dynamic immersion resulted in relatively small but measurable weight variations, reflecting the combined contribution of fluid penetration, medium–polymer interactions, and early material modification processes. The differences observed between static and dynamic conditions further demonstrate the influence of fluid movement and mass transport on the evolving scaffold–medium interface. These findings support the view of the printed scaffold as a dynamic biomaterial microenvironment rather than a static structural element, an important consideration for biomimetic scaffold design and future localized bioactive compound delivery strategies.

The combined biological and material findings suggest that 3D-printed PLA scaffolds may provide a suitable framework for future biomimetic bioactive compound delivery strategies. The temporal differences observed between naringin and hesperidin responses, together with the progressive evolution of the scaffold–medium interface, support the concept of designing delivery systems capable of providing temporally distinct biochemical cues to cells. However, the present study did not evaluate compound loading, adsorption, encapsulation efficiency, or release kinetics. Therefore, the proposed application of PLA scaffolds as bioactive compound delivery platforms remains prospective and requires dedicated experimental validation using bioactive compound-loaded scaffolds and direct release studies.

Despite providing an integrated human cell-based evaluation of bioactive flavonoids together with scaffold–medium interaction analysis, this study has several limitations. Direct drug-loading and controlled-release experiments were not performed, and the scaffold evaluation was limited to short-term immersion and early material evolution. Longer-term physicochemical degradation studies and in vivo validation are therefore required to further establish the potential of the proposed biomimetic delivery strategy.

Overall, the present findings provide complementary human-cell-based and material-level evidence on the temporal responses of WJ-MSCs to naringin and hesperidin and the evolution of 3D-printed PLA scaffolds under static and dynamic immersion conditions. Although these two experimental components were evaluated in parallel rather than within a directly coupled cell–scaffold system, their combined consideration provides a basis for future studies integrating bioactive compounds, human cells, and dynamically evolving scaffolds within a unified experimental platform.

## Figures and Tables

**Table 1:** Percentage weight variation of 3D-printed PLA scaffolds after 1, 3, 7, and 10 days of immersion under static and dynamic conditions.

Time (Days)	% Weight Variation Static Experiment	% Weight Variation Dynamic Experiment
0	0	0
1	−0.343	−0.215
3	−0.247	−0.221
7	−0.299	0.127
10	−0.498	−0.384

**Table 2:** Pairing of events taking place at different moments in time in the cell culture and in the scaffold degradation.

**Day 0**	
*Dynamic PLA scaffold state*	Intact printed architecture; defined surface topography
*Dominant scaffold–medium process*	Initial material state
*Cellular biomarker state*	Baseline
*Naringin* vs. *hesperidin interpretation*	No treatment-specific temporal divergence established
*Biomimetic implication*	Reference state
**Day 1**	
*Dynamic PLA scaffold state*	Early filament breakage; −0.215% mass variation
*Dominant scaffold–medium process*	Initial medium interaction and surface modification
*Cellular biomarker state*	Early ALP-related cellular response
*Naringin* vs. *hesperidin interpretation*	Naringin begins to influence early cellular behavior; hesperidin response remains less pronounced
*Biomimetic implication*	Material–medium interaction begins before substantial bulk mass change
**Day 3**	
*Dynamic PLA scaffold state*	Continued slight mass loss (−0.221%); increasing interaction with surrounding medium
*Dominant scaffold–medium process*	Increasing scaffold–medium interaction, with fluid penetration/retention and material loss potentially occurring concurrently
*Cellular biomarker state*	Early-to-intermediate biomarker transition
*Naringin* vs. *hesperidin interpretation*	Cellular responses begin developing toward later divergence
*Biomimetic implication*	Potential transition point between initial material interaction and stronger biological modulation
**Day 7**	
*Dynamic PLA scaffold state*	Transient positive mass variation (+0.127%); progressive structural modification
*Dominant scaffold–medium process*	Retention of medium-derived components may temporarily offset material loss
*Cellular biomarker state*	ALP/TP and OPN responses reach prominent intermediate-stage values
*Naringin* vs. *hesperidin interpretation*	Naringin shows stronger intermediate response; hesperidin also exhibits substantial biomarker activity
*Biomimetic implication*	Potential temporal coupling window between evolving scaffold–medium interactions and cellular signaling
**Day 10**	
*Dynamic PLA scaffold state*	Pronounced SEM-visible deterioration; −0.384% mass variation
*Dominant scaffold–medium process*	Material loss again predominates over retention
*Cellular biomarker state*	Later-stage biomarker divergence
*Naringin* vs. *hesperidin interpretation*	Naringin response decreases from intermediate peak; hesperidin maintains comparatively stronger late ALP/OPN-related response
***Biomimetic implication***	Potential temporal programming of bioactive delivery, with early/intermediate naringin exposure potentially supporting osteogenic activation and subsequent hesperidin availability potentially supporting sustained cellular and matrix-associated activity
Overall	
*Dynamic PLA scaffold state*	Dynamic evolution rather than static degradation
*Dominant scaffold–medium process*	Competing retention, transport and material-loss processes
*Cellular biomarker state*	Time-dependent and compound-specific cellular trajectories
*Naringin* vs. *hesperidin interpretation*	Early/intermediate naringin response vs. more sustained hesperidin response
*Biomimetic implication*	Provides a rationale for future temporally programmed biomimetic delivery systems

## Data Availability

The datasets generated and/or analyzed during the current study are available from the corresponding author upon request.
